# Two-Step Spark Plasma Sintering Process of Ultrafine Grained WC-12Co-0.2VC Cemented Carbide

**DOI:** 10.3390/ma12152443

**Published:** 2019-07-31

**Authors:** Zhenhua Wang, Jiheng Jia, Boxiang Wang, Yulin Wang

**Affiliations:** School of Mechanical Engineering, Nanjing University of Science and Technology, Nanjing 210094, China

**Keywords:** spark plasma sintering, two-step, ultrafine grain, cemented carbide, relative density

## Abstract

Ultrafine grained WC-12Co-0.2VC (named UYG12V) cemented carbides were prepared via the two-step spark plasma sintering (SPS) in this study. First, the effects of the sintering temperature on the relative density and WC grain size of UYG12V cemented carbides were studied. The results show that regular WC grains form when sintered at 1300 °C. The sintered body begins to rapidly densify and WC grains grow slowly when sintered at 1200 °C. Thus, the first-step (T1) and the second-step (T2) temperatures in the two-step SPS of UYG12V are 1300 °C and 1200 °C, respectively. The effect of the holding time during the first and second steps on the mechanical properties was also studied. The results show that the UYG12V cemented carbide sintered at 1300 °C for 3 min and then at 1200 °C for 5 min has the best comprehensive mechanical properties, exhibiting the average particle size, Vickers hardness, fracture toughness, relative density, and bending strength of 271 nm, 18.06 GPa, 12.25 MPa m^1/2^, 99.49%, and 1960 MPa, respectively.

## 1. Introduction

Due to its high hardness, high wear resistance, stability at high temperature, and other mechanical properties, cemented carbide is widely used in cutting, drilling, and some mining equipment and components [1]. The hardness and strength of cemented carbides improve as the WC grain size decreases to nanometer levels. Therefore, the ultrafine grained cemented carbide has better mechanical properties compared with ordinary alloys. However, during preparation of the ultrafine grained cemented carbide, crystal grains are more likely to grow during sintering because nano-WC particles have a lower surface energy than ordinary WC particles [2,3,4], and it is difficult to obtain ultrafine WC grains using conventional sintering. At present, these are two main ways to inhibit WC grain growth: Adding grain growth inhibitors and selecting a rapid sintering process [5,6].

Most grain growth inhibitors reported in the literature are transition metal carbides, such as TiC, VC, Cr_3_C_2_, ZrC, NbC, TaC, and HfC [7]. Among the inhibitors reported in the literature, VC has the best inhibitory performance, followed by Cr_3_C_2_ [8]. Song et al. [9] prepared the WC-10 ultrafine grained cemented carbide with less than 120 nm particle size and 98.5% relative density with VC as an inhibitor by spark plasma sintering (SPS) at 1100 °C for 5 min. The hardness and fracture toughness of the cemented carbide are *H*_V_ = 2050 ± 10 kgf mm^–2^ and *K*_IC_ = 14.5 ± 0.5 MPa m^1/2^, respectively. However, the inhibitor preferentially dissolves and continuously precipitates in the Co phase, which changes the wettability and fluidity of the Co phase and the solubility of WC in liquid Co, affecting the density of the sintered body [10,11].

In addition to adding inhibitors, a rapid sintering method and tuning the sintering process can suppress grain growth because the grain size grows as the temperature or holding time increase [12]. Joule heating is produced when a high pulse current passes through the sintered powder, providing a sintering effect during SPS [13]. Compared with other sintering methods, SPS provides rapid heating and can complete sintering in a short time. Therefore, grain growth can be suppressed by reducing the grain growth time.

There have been many studies regarding the one-step SPS. Liu [14], Guo [15] and Yan [16] produced cemented carbide blocks with 2000 Vickers hardness and approximately 11 MPa m^1/2^ fracture toughness. However, rapid crystal grain growth is inevitable during the one-step SPS, where sintering is held for a certain period of time at a specific temperature. In order to restrain the crystal grain growth, one can lower the sintering temperature or shorten the holding time. However, if the sintering temperature is too low or the holding time is too short, the relative density of the sintered sample will decrease [17,18,19,20].

The two aforementioned methods reduce the relative density while suppressing the grain growth, thus the two-step sintering method emerged as the times required. Two-step sintering refers to a process wherein the ceramic is heated to a certain critical density after being heated to a high temperature, and the body is rapidly cooled to a second temperature and held for a long period of time. This process yields ultrafine grained ceramics with high relative density [21]. Yin et al. [22] improved the mechanical properties of ceramics using the two-step SPS compared with the ordinary one-step sintering. In the study of Zheng et al., the fracture toughness of WC-Si_3_N_4_ increased from 9.46 MPa m^1/2^ to 10.94 MPa m^1/2^ using the two-step SPS [23]. Although many studies have focused on the two-step sintering of ceramic cutting tools, research on the two-step sintering of tungsten-cobalt cemented carbides is rare.

Many research results show that WC grains grow as the sintering temperature and holding time increase. The formation of WC grains with regular morphology requires a high sintering temperature, and densification requires a long holding time during sintering of the cemented carbide. Therefore, it is difficult to obtain ultrafine grains and high relative density materials simultaneously with a traditional one-step sintering. During the SPS of the cemented carbide, the temperature required to form WC grains with regular morphology is different than that required for densification. The former requires a higher temperature, while the latter requires a lower temperature corresponding to the slower growth of WC grains. Therefore, a two-step SPS sintering method for the UYG12V cemented carbide is proposed in this paper. First, a higher sintering temperature (first-step temperature T_1_) and shorter holding time (first-step holding time t_1_) are used to rapidly form regular fine WC grains. The sintering temperature is subsequently reduced (second-step temperature T_2_) and held for a longer time (second-step holding time t_2_), allowing the materials to densify further.

In this study, the effect of the temperature on the relative density and grain size during the one-step sintering was first studied in order to determine T_1_ and T_2_. The effects of various t_1_ and t_2_ values on the relative density, microstructure, and mechanical properties of UYG12V cemented carbides were studied, and the optimal values of t_1_ and t_2_ were determined.

## 2. Materials and Methods 

The cemented carbide examined herein is composed of 87.8 wt % WC powder (99.9% purity, 60 nm), 12 wt % Co powder (99.9% purity, 600 nm), and 0.2 wt % VC powder (99.9% purity, 600 nm) produced by Shanghai Chaowei Nanotechnology (Shanghai, China). After weighing, the mixed powder was poured into a conical bottle with absolute ethyl alcohol as a vibration medium for ultrasonic vibration while stirring. After 2 h of ultrasonic vibration, the mixed slurry was placed in a vacuum drying chamber and dried at 115 °C. The powder was ground and sifted with a 100 mesh screen after drying. Finally, the prepared powder was placed in a sealed bag for reserve.

An appropriate amount of mixed powder was weighed in the graphite mold with 15 mm diameter bore, and the mold was brought to 10 MPa pre-pressure and held for 3 min. The prepressed mold was placed in a sintering furnace (LABOX^TM^-650F, Sinter Land INC., Nagaoka, Japan) to be sintered at a 30 MPa load. Finally, the sintered samples were allowed to cool to room temperature.

The sintered samples were polished and their mechanical properties were tested. The scanning electron microscopy (SEM, Quant250FEG, FEI, Hillsboro, OR, USA) was used to examine the microstructure of the material. One hundred grains were randomly measured by the Image-Pro 6.0 software. Density was measured using the Archimedes drainage method. The bending strength was measured using an electronic universal testing machine (UTM5105-G, Hengsisheng, Jinan, China). The Vickers hardness on the polished surface was measured using a Vickers diamond pyramid indenter (MN-9631-130-C, INSIZE, Tianjin, China) with 294 N load and 15 s loading holding time. The fracture toughness (K_IC_) was calculated from the length of cracks generated at the four corners of the indenter during loading:

(1) $$K_{\mathrm{IC}}=0.15\sqrt{\frac{HV_{30}}{\sum L}},$$

where L is the length of the crack in the formula.

There are two samples at each sintering temperature. The relative density and bending strength were measured three times and hardness and fracture toughness were measured five times on each sample. First, a one-step sintering experiment was conducted in order to explore the effect of the temperature on the relative density and grain size. The sintering temperature was 1150, 1200, 1250, 1300, and 1350 °C with 100 °C/min heating rate and 1 min holding time. Once T_1_ and T_2_ were determined, the two-step SPS experiments with the UYG12V cemented carbide were conducted at different t_1_ and t_2_ values. The heating rate from 0 to T_1_ was 100 °C/min, and the cooling rate from T_1_ to T_2_ was 200 °C/min during the two-step sintering experiments. The number of samples, experimental design, and results are shown in Table 1.

## 3. Results and Discussion

### 3.1. Analysis of Relative Density and Microstructure of UYG12V Cemented Carbide at Different Sintering Temperatures

**Figure 1:**
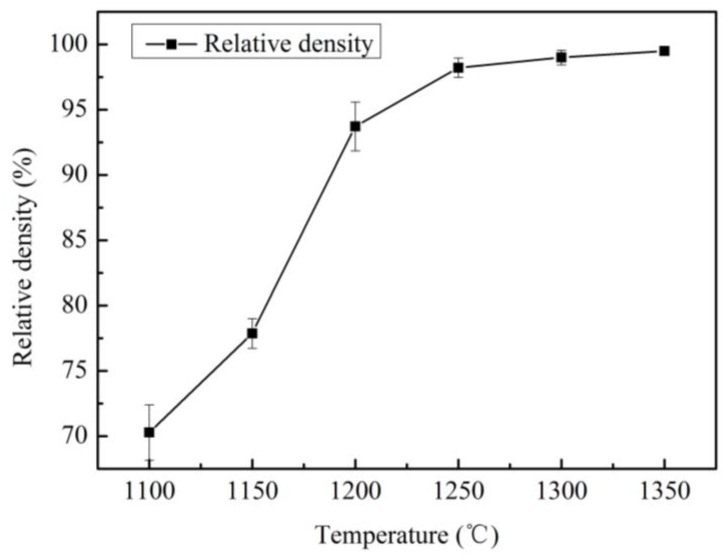
Relationship between relative density and sintering temperature.

The relative density of the UYG12V cemented carbide sintered at different temperatures is shown in Figure 1. One can see in Figure 1 that the relative density of the samples is very low when sintered below 1150 °C, and the relative density increases rapidly from 1150 °C to 1200 °C. The relative density of the sample reaches near 99% when sintered at 1300 °C.

**Figure 2:**
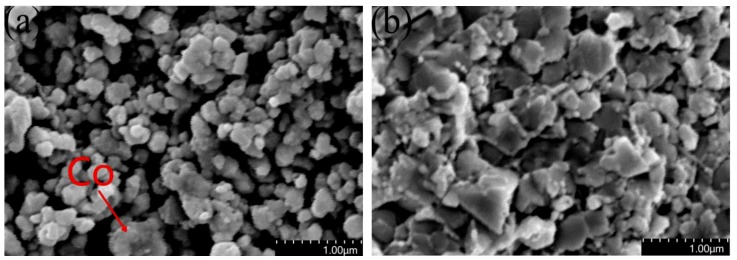
Fracture morphology of the samples sintered at (**a**) 1150 °C and (**b**) 1200 °C.

The fracture morphology (non-corrosive) of the samples sintered at 1150 °C and 1200 °C is shown in Figure 2. Figure 2a shows that there are many gaps in the sample that are sintered at 1150 °C, which reduces the relative density. Some WC grains with granular morphology are bonded to Co. Co liquifies during sintering at 1150 °C and 1200 °C, and WC began dissolving in Co, which marks the beginning of liquid state sintering. The capillary force formed by the liquid phase in the interstitial pore and viscous flow of the liquid phase itself changes the position of the particles and redistributes them to produce the tightest distribution, thus the relative density of the sintered body increases rapidly.

**Figure 3:**
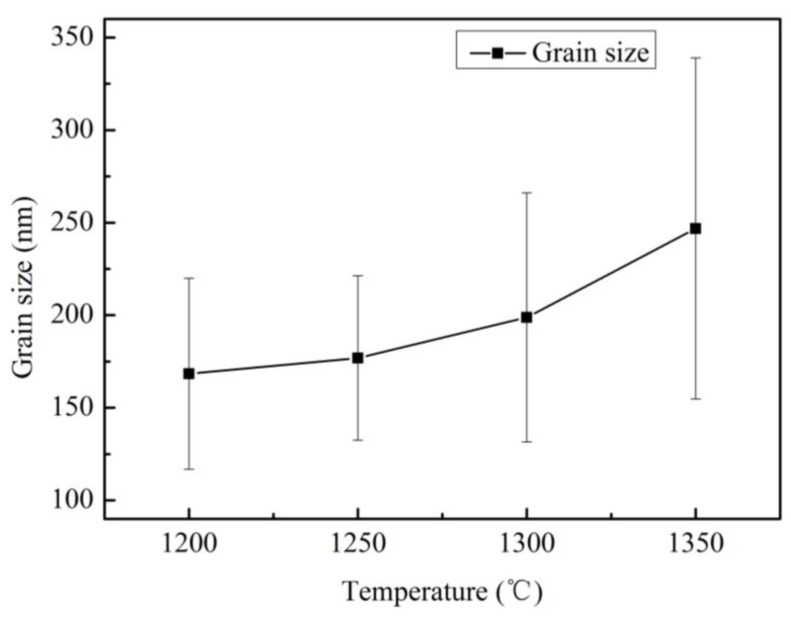
Relation between WC grain size and sintering temperature.

**Figure 4:**
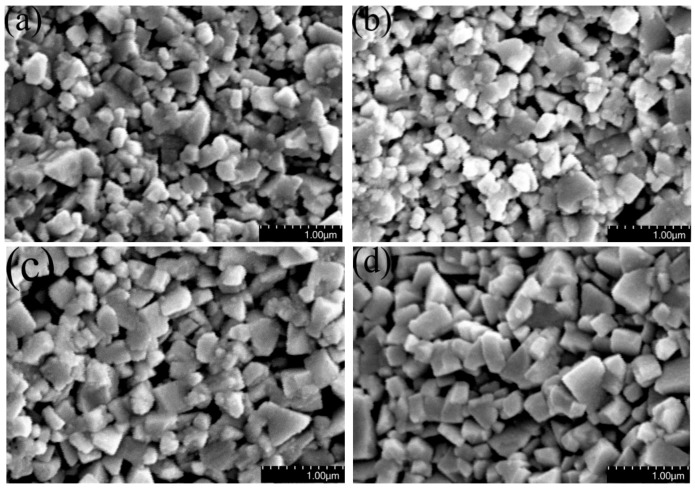
WC grain morphology of samples after holding for 1 min at (**a**) 1200 °C, (**b**) 1250 °C, (**c**) 1300 °C and (**d**) 1350 °C.

Figure 2a WC particles still retain spherical powder shape at 1150 °C due to the solid phase sintering. When the sintering temperature is 1200 °C, the liquid state sintering begins, but dissolution-precipitation of WC in Co is insufficient, and WC grains grow slowly. The relationship between the WC grain size and sintering temperature is shown in Figure 3, wherein one can see that WC grain sizes increase as the sintering temperature increases. This means that grains also grow faster when the sintering temperature exceeds 1250 °C. Most WC grains are irregular blocks that are not conducive to densification, and only a few WC grains reach equilibrium (triangular prism morphology), as shown in Figure 4a. The grain shape does not change much due to sintering at 1250 °C compared with 1200 °C, as shown in Figure 4b. The number of WC grains with regular triangular prism increases significantly at 1300 °C, as shown in Figure 4c. Small, irregular WC grains are present in the samples sintered at 1200 °C and 1300 °C, and the number of irregular WC grains decreases as the temperature increases. Rapid grain growth occurs during sintering at temperatures between 1300 °C and 1350 °C, and the average grain size of WC rapidly increases from 208.8 nm to 246.8 nm as temperature increases, as shown in Figure 3. This occurs because the energy barrier preventing W and C migration increases as the temperature decreases [24]; migration of W and C and dissolution-precipitation of WC in Co proceed at a higher rate at high temperature, which promotes rapid grain growth. At 1350 °C, most WC grains transform into triangular prisms, and nearly all WC particles less than 100 nm disappear, as shown in Figure 4d. Therefore, during the two-step sintering process, T_1_ = 1300 °C was chosen as the first-step temperature in order to form a regular grain morphology. T_2_ = 1200 °C was chosen as the second-step temperature in order to form the liquid phase, thus increasing the relative density of the samples while inhibiting rapid grain growth.

### 3.2. Analysis of Relative Density and Microstructure of Two-Step Sintered UYG12V

**Figure 5:**
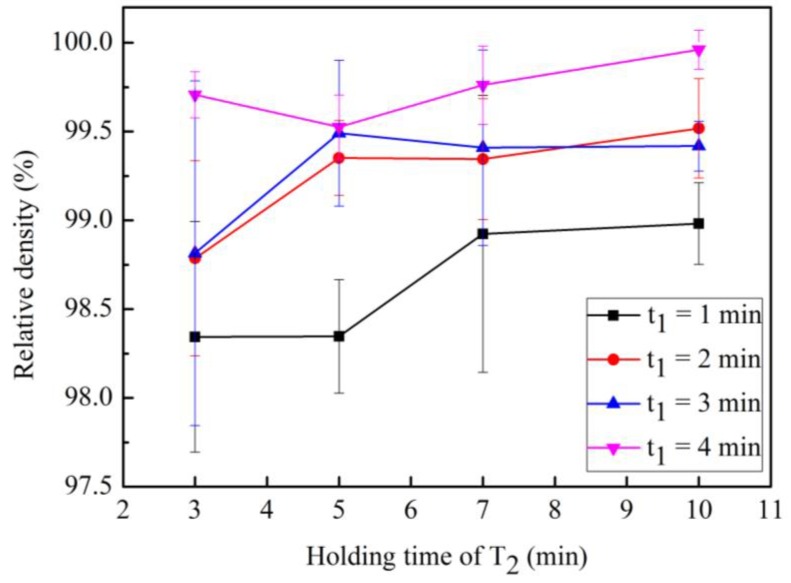
Relationship between sample relative density and holding time.

Figure 5 shows the relationship between the relative density and holding time. Figure 5 shows that the relative density of the samples increases as the holding time increases. In the case where t_1_ = 2 min and 3 min, the relative density of the samples reaches 99.4% when t_2_ = 5 min. In addition, in the case of t_1_ = 1 min, the relative density of the samples reaches 98.9% when t_2_ = 7 min. Thus, one can conclude that t_2_ influences the relative density of the sintered sample. As the extension of t_2_, the liquid phase exists for a longer time when t_2_ is larger, which encourages the rearrangement of WC particles and increases the relative density of the samples.

**Figure 6:**
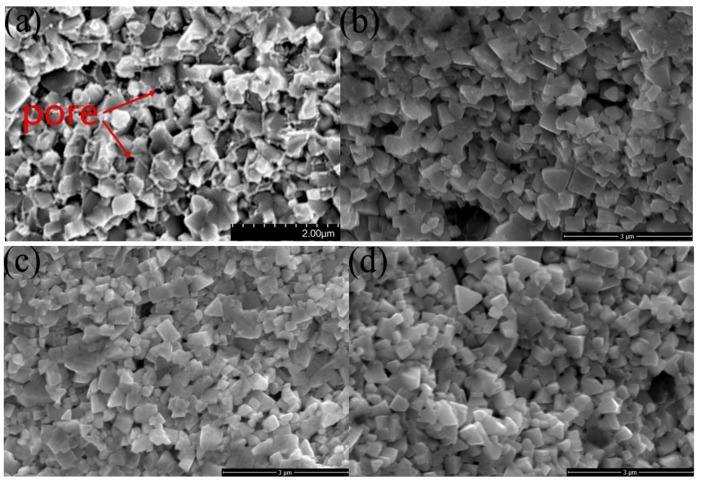
Fracture morphology of (**a**) 1a, (**b**) 1a (no Co), (**c**) 1d (no Co), (**d**) 4a (no Co) sample.

Figure 6 shows the fracture morphology of the sintered UYG12V cemented carbide. When the grain morphology of samples 1a and 1d are compared, one finds that the irregular grain morphology of WC does not change as t_2_ increases. On the contrary, when the grain morphology of samples 1a and 4a are compared, one finds that the grain morphology of WC tends to be more regular as t_1_ increases, which favors grain rearrangement increases the relative density. Therefore, when t_1_ = 1 min, the relative density tends to be low regardless of the value of t_2_. A short holding time at high temperature causes the dissolution-precipitation of WC to occur at a high rate for a short period of time. Dissolution-precipitation will dissolve particles with a large surface curvature, and dissolved substances will precipitate in areas with negative curvature. The shapes of the resulting particles will be more flat, which is more conducive to the rearrangement of particles. Therefore, under the condition that the dissolution-precipitation of WC is suppressed, WC grains have some region with negative curvature and high relative density cannot be achieved only by prolonging the particle rearrangement time. The porosity of sample 1a can be seen in Figure 6a, and the irregular grain morphology of sample 1a can be seen in Figure 6b. One can conclude that t_1_ in determining the morphology, which greatly affects the relative density of the sintered samples. Among all samples, the samples with t_1_ = 4 min have the highest relative density, and changing t_2_ has little effect on the relative density.

**Figure 7:**
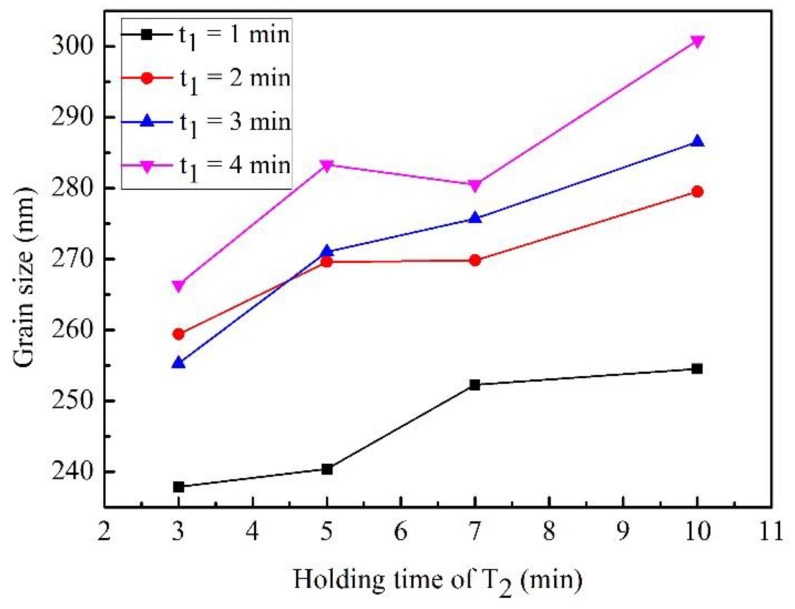
Relationship between the WC grain size and holding time.

Figure 7 shows the relationship between the grain size and holding time, which indicates that the WC grain size increases as the holding time increases. This is inconsistent with the conclusion regarding the relationship between the grain size and holding time provided by Bao et al. [25], who pointed out that the effect of the holding time on the grain size is extremely small, and the grain size does not increase as the holding time increases. This difference may arise because Bao et al. reached this conclusion based on results from microwave sintering, wherein the heating rate is slow and the sintering time is long, in contrast to SPS. There may be a threshold holding time beyond which the grain growth rate becomes very slow. The holding time easily reaches this point during microwave sintering due to the long sintering time. However, unlike microwave sintering, SPS has a fast heating rate and short holding time. Therefore, the grain size is strongly affected by the holding time and grain growth can be controlled by adjusting the holding time in SPS. Smaller grains can be obtained by the two-step SPS than microwave sintering.

In the two-step SPS process, the average growth rate of WC grains is 12.16 nm min^–1^ at T_1_ and 3.67 nm min^–1^ at T_2_. One can see that the effect of the holding time on the WC grain size at high temperature is greater than that at low temperature.

**Figure 8:**
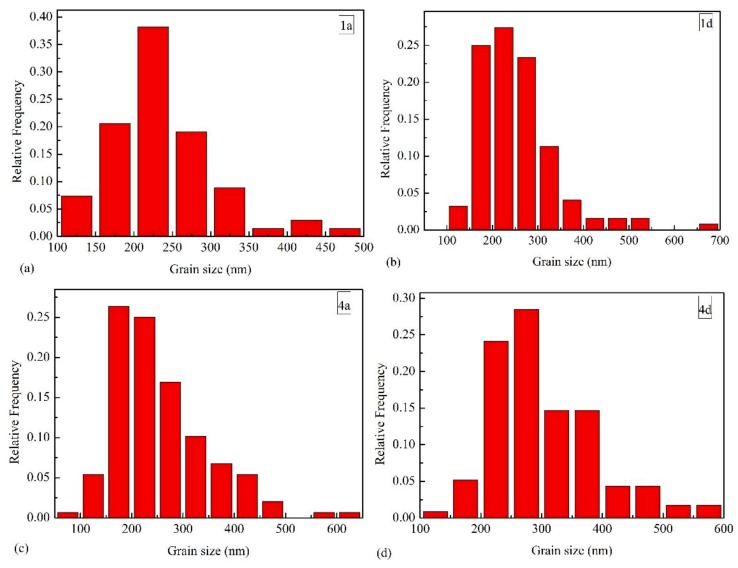
Grain size distribution of (**a**) 1a, (**b**) 1d, (**c**) 4a and (**d**) 4d sample.

Figure 8 shows the effect of the holding time on the WC grain distribution. One can see that the proportion of WC particles with the grain size larger than 500 nm increases as t_1_ or t_2_ increase. This is related to the overall grain growth, which increases the proportion of the large WC during a longer holding time. In general, during the two-step plasma sintering, increasing the holding time has little effect on the grain size distribution and does not cause a sudden increase in the number of abnormal grains.

### 3.3. Analysis of Mechanical Properties during Two-Step Sintering of UYG12V

**Figure 9:**
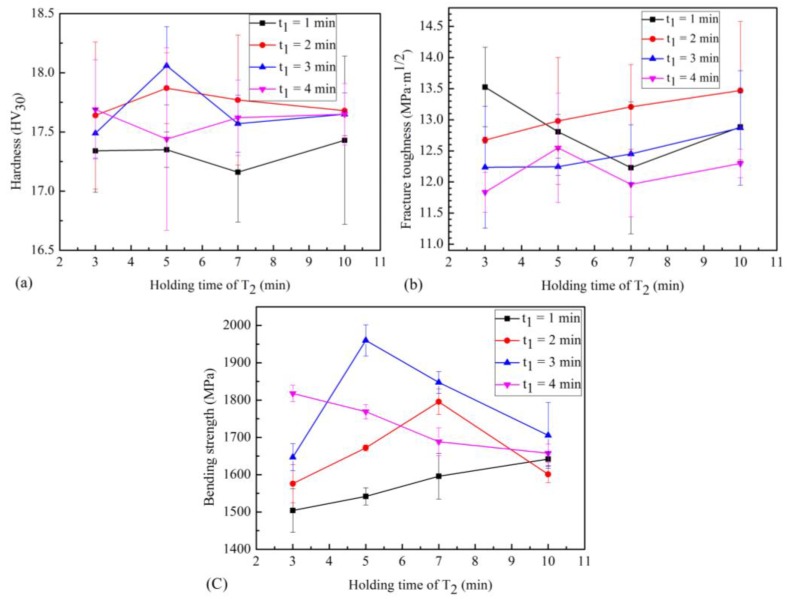
Relationship between the holding time and (**a**) hardness, (**b**) fracture toughness, and (**c**) bending strength.

The hardness of WC-Co cemented carbides tends to increase and then decrease as the sintering temperature or holding time increase. Moreover, the effect of the sintering temperature on the hardness of tungsten-cobalt cemented carbides is greater than the effect of the holding time. Therefore, using a low sintering temperature and short holding time will result in a material with low relative density and many pores, which decreases its hardness. In contrast, the high sintering temperature and long holding time will lead to the abnormal grain growth in WC-Co cemented carbides, which also decreases their hardness. Figure 9 shows the relationship between the selected mechanical properties and holding time. Figure 9a shows that the hardness of samples with high relative density increases first and then decreases as t_2_ increases when t_1_ = 2 or 3 min. In the case where t_1_ = 1 min, the hardness of samples is generally low due to their low relative density. However, in the case where t_1_ = 4 min, the grain size of the samples increases, and increasing t_2_ does not significantly affect their hardness values.

Figure 9b shows the relationship between the fracture toughness and holding time. Compared with samples where t_1_ = 1 or 4 min, one can conclude that the fracture toughness of the samples decreases as the relative density increases. Crack propagation in the ultrafine grained cemented carbide occurs via the intergranular fracture rather than the transgranular fracture, and pores in the cobalt phase (Figure 6a) hinder crack propagation, which helps increase the fracture toughness of the samples [11]. When t_1_ > 1 min for samples with higher relative density, harder samples have lower toughness, which may be related to the size of ultrafine WC particles and the distribution of cobalt. As the WC grain size is smaller, the thickness of the Co layer between the WC particles is smaller. Therefore, in ultrafine grained cemented carbides, the thickness of the Co layer tends to be lower than the critical value corresponding to the brittle Co [26]. 

Among the sixteen samples in our experiments, the hardness of sample 3b was found to be the highest (18.06 GPa), and the fracture toughness is 12.25 MPa m^1/2^. The fracture toughness of sample 1a is the highest (13.53 MPa m^1/2^), and its hardness is 17.34 GPa.

As shown in Figure 9c, the bending strength of the sintered samples increases and then decreases as t_2_ increases when t_1_ = 2 or 3 min. Meanwhile, the bending strength of the samples decreases as t_2_ increases when t_1_ = 4 min. Finally, the bending strength of the samples increases as t_2_ increases when t_1_ = 1 min. Such changes are closely related to the low relative density of the samples caused by the short holding time and grain growth caused by the long holding time. Pores or grown grains maybe concentrate stress and become the starting points of crack propagation or fracture, thus decreasing the bending strength.

**Figure 10:**
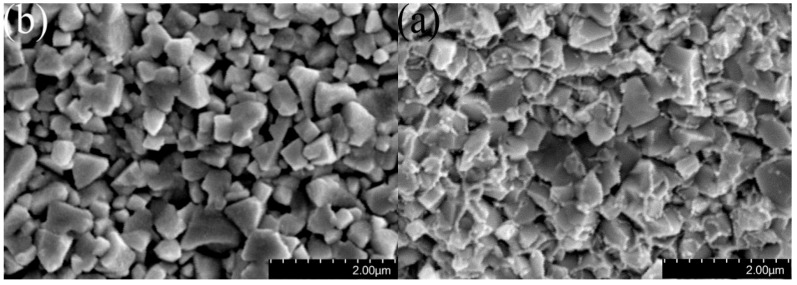
SEM image showing (**a**) containing Co and (**b**) no Co fracture morphology of 3b.

In summary, during the two-step sintering process, sample 3b exhibits the highest hardness and bending strength with no obvious porosity and relatively uniform WC grain size (Figure 10). The average WC grain size of the ultrafine grained cemented carbides decreased from 362 nm to 271 nm, and the relative density increased from 98.6% to 99.5%, while the hardness increased from 17.79 GPa to18.06 GPa and the bending strength increased from 1899 MPa to 1960 MPa, compared with the ultrafine grained cemented carbides prepared by Liu et al. using one-step sintering [27].

## 4. Conclusions

In this paper, UYG12V cemented carbides were prepared using the two-step SPS, and their relative densities, grain sizes, and mechanical properties were studied. During sintering, the liquid phase appears and the relative density of the sample increases rapidly at sintering temperatures ranging from between 1150 °C to 1200 °C, during which the WC grains grow slowly. Increasing t_1_ or t_2_ increases the relative density, particularly t_1_. The WC grain size of the cemented carbide is related to the holding time and sintering temperature, and the grain size increases as the holding time or sintering temperature increases. In addition, t_1_ is a stronger determinant of the grain size than t_2_ and plays a decisive role in determining the WC grain shape. UYG12V sintered at 1300 °C for 3 min and 1200 °C for 5 min exhibit an average particle size, Vickers hardness, fracture toughness, relative density, and bending strength of 271 nm, 18.06 GPa, 12.25 MPa m^1/2^, 99.49% and 1960 MPa, respectively.

## Figures and Tables

**Table 1:** Number of samples, experimental design, and results.

Number of Samples	t_1_ (min)	t_2_ (min)	Relative Density (%)	Grain Size (nm)	Hardness (GPa)	Fracture Toughness (MPa m^1/2^)	Bending Strength (MPa)
1a	1	3	98.34	237.9	17.34	13.53	1504
1b	1	5	98.35	240.4	17.35	12.81	1542
1c	1	7	98.92	252.3	17.16	12.23	1596
1d	1	10	98.98	254.5	17.43	12.88	1642
2a	2	3	98.79	259.4	17.64	12.67	1576
2b	2	5	99.35	269.6	17.87	12.98	1672
2c	2	7	99.35	269.8	17.77	13.21	1796
2d	2	10	99.52	279.5	17.68	13.47	1601
3a	3	3	98.82	255.3	17.49	12.24	1647
3b	3	5	99.49	271.0	18.06	12.25	1960
3c	3	7	99.41	275.7	17.57	12.45	1848
3d	3	10	99.42	286.5	17.65	12.87	1706
4a	4	3	99.71	266.4	17.69	11.84	1818
4b	4	5	99.53	283.3	17.44	12.55	1769
4c	4	7	99.76	280.5	17.62	11.96	1689
4d	4	10	99.96	300.8	17.65	12.30	1658
